# Does *Porphyromonas gingivalis* truly inhibit the oral carcinogenesis?

**DOI:** 10.1002/1878-0261.70037

**Published:** 2025-04-25

**Authors:** Chen‐xi Li, Zhong‐cheng Gong

**Affiliations:** ^1^ Department of Oral and Maxillofacial Oncology & Surgery, The First Affiliated Hospital of Xinjiang Medical University, School/Hospital of Stomatology Stomatological Research Institute of Xinjiang Uygur Autonomous Region Urumqi China; ^2^ Precision Medicine Key Laboratory of Sichuan Province West China Hospital of Sichuan University Chengdu China

The oral microbiome population varies by saliva and different habitats of the oral cavity. Tobacco smoking, alcohol consumption, and betel nut chewing, which are causative factors of oral cancer, may alter the oral microbiome composition. Over the past several decades, accumulating evidence has suggested that both pathogenic and commensal strains of bacteria have significantly contributed to oral squamous cell carcinoma (OSCC) [1]. Today, the mainstream belief in academic circles can be divided into two aspects: (a) numerous bacterial species, including bacterial products and their metabolic by‐products released in the mouth, are involved in chronic inflammation that leads to the progression of oral carcinogenesis, and (b) the intratumoral host‐microbiota, as an intrinsic component of the tumor microenvironment (TME) across oral cancer, may induce permanent genetic alterations in the epithelial cells of the host that drive proliferation and/or survival of epithelial cells [2, 3]. These studies have resulted in the hypothesis that the inflammatory microbiota associated with periodontitis may participate in the development and progression of OSCC.

Increasing evidence demonstrates that *Porphyromonas gingivalis* (*P. gingivalis*)—one of classical periodontopathogens—plays a critical role in the formation and progression of OSCC [4]. Moreover, these organisms share the ability to attach and invade oral epithelial cells, and from there each undergoes its own unique molecular dialogue with the host epithelium, which ultimately converges on acquired phenotypes associated with cancer, including inhibition of apoptosis, increased proliferation, and activation of epithelial‐to‐mesenchymal transition (EMT) leading to increased migration of epithelial cells [1, 4]. Products and its metabolic by‐products of *P. gingivalis* may induce permanent genetic alterations in epithelial cells of the host that drive immune escape and survival of OSCC cells, and inhibit the cytotoxicity of programmed cell death [5]. In addition, virulence factors (gingipains, capsule, fimbriae, hemagglutinins, lipopolysaccharide, hemolysin, iron uptake transporters, toxic outer membrane blebs/vesicles, and DNA) associated with *P. gingivalis* can deregulate certain functions in humans, particularly host immune systems, and cause various local and systemic pathologies including cerebral, cardiovascular, pulmonary, bone, digestive, and peri‐natal infections [1, 5, 6]. These biomechanisms fully reflect that *P. gingivalis* is not considered as a sort of ‘probiotics’.

Recently, we read with great interest the publication of Lan and colleagues. Their findings that *P. gingivalis* successfully reversed the immunosuppressive TME, thereby suppressing the growth of OSCC through the MUC1/CXCL17 signaling axis, indicating the rational use of *P. gingivalis* could serve as a promising therapeutic strategy for OSCC [7]. However, we believe some issues regarding this study did not appropriately support this conclusion. First, given the well‐known tumor‐promoting action of *P. gingivalis*, a commensal bacterium (such as *Veillonella parvula*) as non‐carcinogenic control strain was absent. Second, it is necessary to ensure the elimination of potential tumor‐resident microbes before the establishment of tumor‐bearing mice to better prove the independent role of *P. gingivalis* impeding primary tumor growth. Third, MUC1‐knockout mice models should be used to deeply verify murine oral carcinogenesis involving a CXCL17‐mediated pro‐tumorigenic immune cells recruitment feedback loop. Accordingly, the findings on ‘stifling effect to OSCC resulted from *P. gingivalis*’ remain pending. Furthermore, authors mentioned that multiplicity of infection (MOI) of coculture was set as 0, 1, 10, 100, and 1000 and without any sound and valid references to support and explain these sets of different concentrations of *P. gingivalis*; since generally MOI is 10–50 (no more than 200 for coculture with OSCC cells). Predominantly, any high concentration of bacteria has a killing effect on cancer cells because these bacteria will exhaust a large amount of energy resulting in cell death. Additionally, to control for false positives due to possible cross‐reaction of the antibody, the quantitative analysis of *P. gingivalis* in surgical specimens could be evaluated by real‐time PCR. It would be important to confirm by PCR the presence of *P. gingivalis* DNA in the samples positive for immunohistochemistry. Another fatal flaw designed in survival analysis for patients with primary OSCC is lack of control samples from *P. gingivalis*‐negative OSCC patients. These reasons may lead to the ‘irregularity’ reported by Lan et al. Our investigation containing 205 patients with OSCC discovered that the high immunoexpression level of *P. gingivalis* has an enhanced risk with lower 10‐year cumulative survival rate (CSR) compared with patients with low immunoexpression level of *P. gingivalis* and patients with negatively expressed OSCC (Fig. 1). This result is in line with the available literature, but is adverse to the currently commented study.

**Fig. 1:**
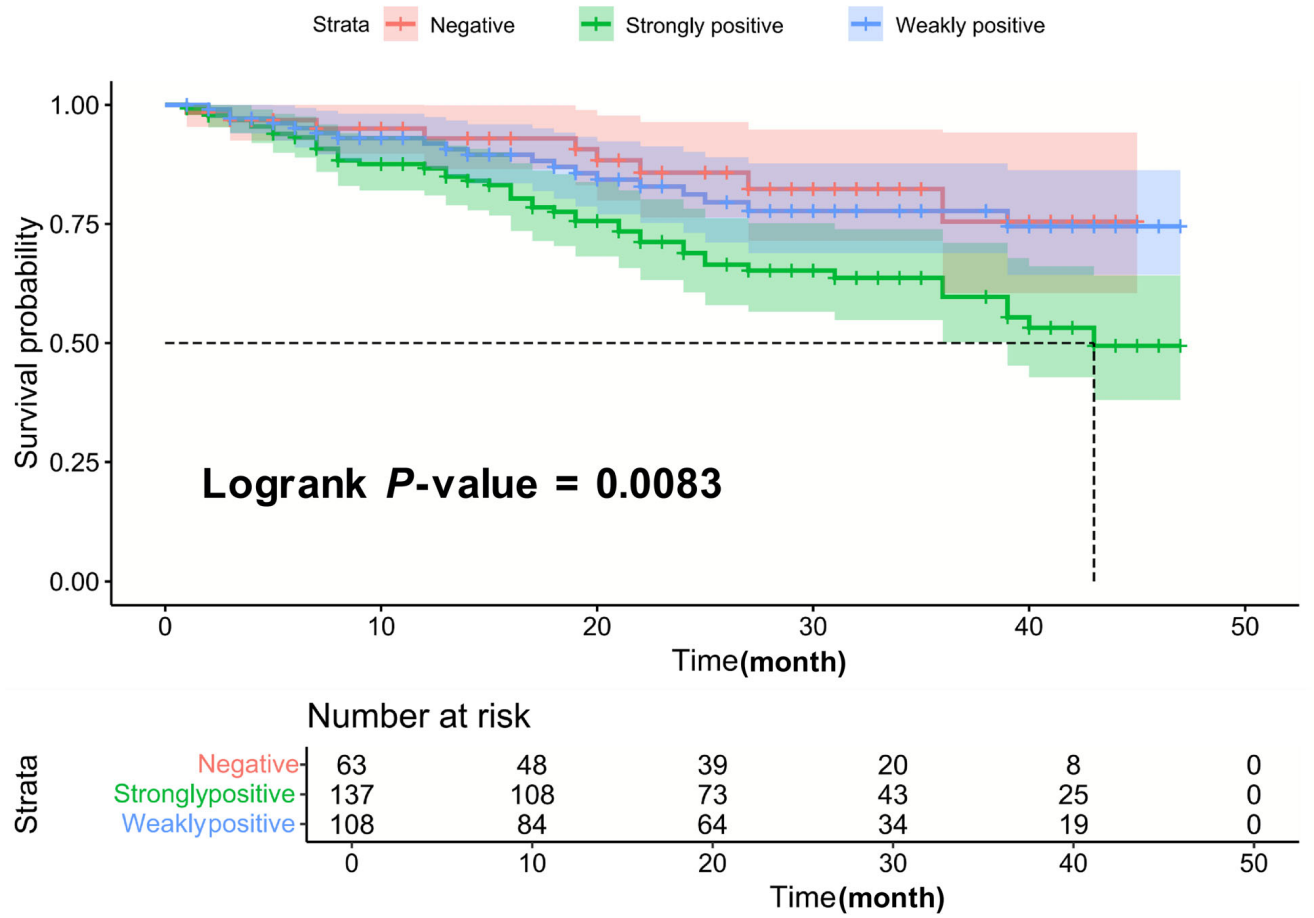
Prognosis of oral squamous cell carcinoma (OSCC) that was associated with the abundance of *Porphyromonas gingivalis*. Quantification for *P. gingivalis* is determined through immunohistochemistry results for the presence of *P. gingivalis* in the samples by PCR. The Kaplan–Meier approach and log‐rank test were used to plot the survival curves [4].

Collectively, oral and orodigestive cancers harbor a diverse microbiome that differs compositionally from precancerous and healthy tissues. Though causality is yet to be definitively established, emerging trends implicate periodontal pathogens such as *P. gingivalis* as being associated with the cancerous state. Moreover, infection with *P. gingivalis* correlates with a poor prognosis (Fig. 1), and *P. gingivalis* is oncopathogenic in animal models [8]. Mechanistically, properties of *P. gingivalis* that have been established *in vitro* and could promote tumor development include induction of a dysbiotic inflammatory microenvironment, inhibition of apoptosis, increased cell proliferation, enhanced angiogenesis, activation of EMT, and production of carcinogenic metabolites [1, 5, 6]. The microbial community context is also relevant to oncopathogenicity, and consortia of *P. gingivalis* and *Fusobacterium nucleatum* are synergistically pathogenic in oral cancer models *in vivo* [1, 9]. Although Lan et al. provided unprecedented findings identifying *P. gingivalis* as a new bioagent for the treatment of OSCC, its role in the OSCC tumor immunomicroenvironment is well worth further investigation.

## Conflict of interest

The authors declare no conflict of interest.

## Author contributions

CL wrote the original draft and revised the manuscript. ZG reviewed the manuscript.
